# A small dose of dezocine suppresses remifentanil-induced cough in general anesthesia induction: a prospective, randomized, controlled study

**DOI:** 10.1186/s12871-020-01156-x

**Published:** 2020-09-16

**Authors:** Rui Ma, Yu Wei, Zifeng Xu

**Affiliations:** 1 Department of Anesthesiology, international Peace Maternal and Child Health Hospital, School of Medicine, Shanghai Key Laboratory of Embryo Original Disease, Shanghai Municipal Key Clinical Specialty, Shanghai Jiao Tong University, Huashan Rd. 1961, Shanghai, 200030 China; 2 Shanghai Key Laboratory of Embryo Original Diseases, Shanghai, China

**Keywords:** Opioids, Cough, General anesthesia, Dezocine, Remifentanil

## Abstract

**Background:**

The aim of this prospective randomized controlled study was to evaluate whether pretreatment with a small dose of dezocine could prevent remifentanil-induced cough in general anesthesia induction.

**Trial design:**

a prospective, randomized, controlled study.

**Methods:**

A total of 210 patients receiving elective operative hysteroscopy from December 2018 to April 2019 were enrolled in the present study. They were randomly equally separated into dezocine group (*n* = 105) and control group (*n* = 105). Patients were intravenously pre-administrated with dezocine 0.03 mg/kg (diluted to 5 mL) or the same volume of normal saline 1 min prior to remifentanil infusion. One minute later, intravenous injection of propofol 1.5 mg/kg and cisatracurium 0.1 mg/kg were given to all patients for induction of general anesthesia. The counts of coughs occurred during the anesthesia induction period were recorded and the severity of cough was scaled.

**Results:**

There were 7 cases of mild cough in dezocine group and 18 cases of mild cough, 12 cases of moderate cough and 4 cases of severe cough in control group. The incidence rate of cough was significantly lower and the severity of cough was obviously relieved in dezocine group compared to control group (6.67% vs. 32.38%, *P* <  0.001). The two groups were not significantly different in heart rate and mean arterial pressure before the induction, before and after the intubation, and in operating time and postoperative visual analog scale pain scores.

**Conclusion:**

This study recommends the efficacy and safety of a pretreatment with a small dose of dezocine in reducing remifentanil-induced cough during general anesthesia.

**Trial Registration:**

ChiCTR2000032035.

Date of registration: Retrospectively registered on 2020/04/18.

## Background

Opioids are conventionally used as adjunct drugs for induction of general anesthesia [1]. Application of opioids such as fentanyl [2] and sufentanil [3] often elicits cough during induction of general anesthesia. This adverse effect may lead to increased intracranial pressure and intraocular pressure and is extraordinarily dangerous for patients with diseases such as cerebral aneurysm, open eye injury and airway [4]. Remifentanil is a fentanyl congener characterized by short onset and short action time, and has been widely applied for induction of general anesthesia [5]. Remifentanil shows an antitussive effect on brainstem μ-opioid receptors [6]; however, some studies reported it also causes coughing just like fentanyl with a incidence rate varies from 26 to 57% during general anesthesia [7–10].

There is a growing interest in suitable agents and methods to prevent remifentanil-induced cough. For instance, Kim et al. suggests that elongating injection time over than 60 s could inhibit remifentanil-cough during anesthesia in children [7]. Limited maximal infusion rate appears to alleviate remifentanil-induced cough as well [11]. Moreover, there is evidence that pretreatment of several agents such as dexamethasone [12], ketamine or their combination [13] and propofol [14] has been reported to be useful for suppressing cough provoked by remifentanil. Dezocine is a mixed agonist-antagonist opioid analgesic [15]. It was initially identified as a full agonist of κ-receptor and partial agonist/antagonist of μ-receptor [15]. Later study suggested it is a κ-receptor antagonist [15, 16]. Increasing studies have demonstrated that dezocine administration is effective and feasible in preventing the occurrence of sufentanil-induced cough during the induction of general anesthesia [17, 18]. Nevertheless, few studies are focused on the effect of dezocine on remifentanil-induced cough.

Our study pretreated the patients undergoing elective hysteroscopic surgery with a small dose of dezocine prior to remifentanil infusion with a view to investigating whether dezocine pretreatment is a reliable strategy to relieve the incidence and severity of remifentanil-induced cough. This prospective randomized controlled trial would provide some useful clues for further development of anesthesia management.

## Methods

### Study design

This study was approved by the National science and technology ethics committee (approval number: 2017–101) and registered in the Chinese Clinical Trial Register (registration number: ChiCTR2000032035). A total of 210 patients with ASA physical status I-II who received elective hysteroscopic surgery in our hospital from December 2018 to April 2019 were enrolled in this study. Exclusion criteria were: known hypersensitivity to the drugs used in this study; body mass index > 30 kg/m^2^; a history of hypertension; severe arrhythmias; chronic bronchitis, asthma, or other respiratory diseases; increased intraocular pressure, intrathoracic pressure or intracranial pressure before surgery. Written informed consent was obtained from each participant.

The incidence of remifentanil (1.5 μg/kg)-induced cough in control group was around 30% in our early study (unpublished). We hypothesized that the incidence rate of cough in dezocine group was lower than 10%. Based on α = 0.05 (two-sided) and power = 0.95 (β = 0.05), a medium effect size (Cohen’s f = 0.25), the estimated sample size was 104. Taking the dropout into account, the sample size was set at 105 in each group.

The 210 patients were randomly and equally divided into two groups: dezocine group and control group by a computer-generated allocation program.

### Anesthesia and monitoring

In brief, all patients of the two groups were fasted for 8 h. Peripheral venous access was established using a 20-gauge cannula on the dorsal hand in the operating room. Heart rate (HR) and blood pressure were monitored. Two minutes prior to induction of general anesthesia, patients of the dezocine group received intravenous infusion of dezocine 0.03 mg/kg (diluted to 5 mL), while patients of the control group received an injection of 5 mL normal saline.

One minute later, remifentanil 1.5 μg/kg (1 mg remifentanil diluted with normal saline in a 50 mL syringe; Yichang Humanwell Pharmaceutical Co., LTD, Yichang, Hubei Province, China) was administrated to all patients of the two groups by a syringe pump at a rate of 600 mL/h, followed by intravenous injection of propofol 1.5 mg/kg and cisatracurium 0.1 mg/kg (Hengrui Pharmacutical Co., LTD, Shanghai, China) 1 min later to induce general anesthesia. Endotracheal intubation was then conducted and the patients were ventilated mechanically.

HR and mean arterial pressure (MAP) before the anesthesia induction, immediately before and 1 min after intubation were recorded. Side effects such as vomiting [19], hypoxemia (SpO_2_ < 90%) [20] or other intended effects during the induction period were also recorded. The number of coughs of the two groups was recorded and the severity of cough was defined as follows: mild, 1–2; moderate, 3–4; severe, 5 or more [21, 22]. In addition, operative time and visual analog scale (VAS) pain score 30 mins and 60 mins postoperatively were recorded and analyzed as well.

### Statistical analysis

The primary endpoint was the incidence of cough in different groups. All statistical analyses were done by SPSS software 22.0 (IBM, Chicago, IL, USA). Categorical data, such as ASA and incidence of cough were expressed as number (percentage) and compared between groups by Chi-square test or Wilcoxon rank-sum test. Continuous data, including age and body weight were expressed as mean ± standard deviation and compared by Students’ t test. Repeated continuous variables, including HR, MAP were analyzed by repeated measure ANOVA with Bonferroni correction. *P* <  0.05 was defined as significance level.

## Results

### Demographic characteristics

Total 210 ASA I and II patients were included in this study. They were randomly assigned to dozocine group (*n* = 105) or control group (*n* = 105). The age range of patients in dozocine group was 25–73 years and body weight range was 43–80 kg. The age range and body weight of patients in control group were 22–75 years and 42–89 kg, respectively. There was no significant difference between the two groups in age (41.99 ± 10.43 years vs. 42.61 ± 11.26 years, *P* = 0.680), body weight (58.82 ± 7.92 kg vs.59.50 ± 9.93 kg, *P* = 0.586) and ASA class (*P* = 0.644) (Table 1).

**Table 1:** Demographic characteristics of patients in two groups

Group	Control group	Dezocine group	*P*
n	105	105	
Age (year)	41.99 ± 10.43	42.61 ± 11.26	0.680
Body weight (kg)	58.82 ± 7.92	59.50 ± 9.93	0.586
ASA class (I/II)	78/27	74/31	0.644

Data represent mean ± SD or numbers. Difference between groups were compared by students’ t test or chi-square test

Hysteroscopic surgery was successful in all patients. Intraoperative oxygen saturation (SpO_2_) of each patient was 100%. Adverse effects such as nausea and vomiting were not observed in any patient. HR and MAP were not significantly different between the two groups before the anesthesia induction, before intubation and 1 min after intubation (*P* > 0.05, Table 2). Moreover, both the two groups had significantly decreased HR and MAP before intubation and 1 min after intubation compared to those before the anesthesia induction (*P* < 0.05, Table 2).

**Table 2:** Observations of MAP and HR

Variable	Group	Before induction	1 min before intubation	1 min after intubation
(mmHg)	Control group	85.64 ± 8.32	70.26 ± 7.47*	69.79 ± 6.19*
	Dezocine group	88.03 ± 14.91	70.06 ± 8.24*	70.07 ± 7.94*
HR (beats/min)	Control group	81.77 ± 13.04	60.86 ± 8.04*	60.28 ± 7.81*
	Dezocine group	79.58 ± 13.71	60.89 ± 7.55*	60.19 ± 6.69*

Difference between groups at same time point was compared by Student’s t test. Difference among different time points in the same group was compared by repeated measure ANOVA. *P < 0.05 vs. before anesthesia induction. MAP, mean arterial pressure; HR, heart rate

### Incidence and severity of cough, operating time and VAS pain score

As shown in Table 3, mild cough was observed in 7 patients of dozocine group, with an incidence of 6.67%. Control group had 34 patients with cough (32.38%), including 18 patients with mild cough, 12 patients with moderate cough and 4 patients with severe cough. The patients in dozocine group had a significantly lower incidence of cough and improved severity of cough in comparison with those in control group (*P* < 0.001). Additionally, there were no significant differences in operating time, 30-min and 60-min postoperative VAS pain scores (*P* > 0.05, Table 4).

**Table 3:** Incidence and severity of cough in two groups

Group	N	None (N/%)	Mild cough (N/%)	Moderate cough (N/%)	Severe cough (N/%)	Cough (N/%)	P
Control group	105	71(67.62%)	18(17.31%)	12(11.43%)	4(3.81%)	34(32.38%)	< 0.001
Dezocine group	105	98(93.33%)	7(6.67%)	0	0	7(6.67%)

**Table 4:** Operative duration and postoperative VAS pain scores of two groups

Group	Operative time (min)	30-min postoperative VAS pain scores	60-min postoperative VAS pain scores
Group C	20.4 ± 3.5	2.83 ± 1.33	3.81 ± 1.12
Group D	19.8 ± 4.3	2.65 ± 0.97	3.79 ± 1.11

VAS Visual analog scale

## Discussion

Our study enrolled a total of 210 patients scheduled for hysteroscopic surgery who were equally randomized into dozocine group and control group. Patients in these two groups had similar age and body weight. The patients in the dozocine group received a small dose of dezocine prior to remifentanil administration, while patients in control group were given the same volume of normal saline. A pre-emptive administration of dezocine decreased the incidence of cough from 32.38 to 6.67% and relieved the severity of cough. These findings suggest that pretreatment with dezocine could effectively prohibit remifentanil-induced cough during general anesthesia induction in patients undergoing operative hysteroscopy.

Hysteroscopy is an established endoscopic surgical procedure in obstetrics and gynecology practice for diagnosis and management of endometrial problems, with advantages of fewer complications, shorter recovery time and lower costs [23, 24]. However, pain is the most common complication of hysteroscopy because of several procedures such as cervical dilatation, uterine distension and peritoneal irritation [25]. Application of appropriate anesthetic agents for maximal anesthetic effect is a critical issue for hysteroscopy [26]. Remifentanil is a short-acting anilidopiperidine opioid with unique pharmacokinetic characteristics, facilitating fast and efficient analgesia. It is metabolized by esterases independently of hepatic and renal functions, and was therefore eliminated at a more rapid speed than other anilidopiperidine opioids [27]. Remifentanil alone or in combination with adjunct agents has been widely utilized for general anesthesia in hysteroscopy [28]. In the present study, similarly, intravenous infusion of remifentanil 1.5 μg/kg in synergy with propofol 1.5 mg/kg and cisatracurium 0.1 mg/kg 1 min later were used for the induction of general anesthesia in all patients. Cough is often elicited by remifentanil infusion and is a causative factor of undesirable conditions including elevated intracranial, intra-ocular and intra-abdominal pressure [29]. In the present study, the incidence of remifentanil-induced cough without any pretreatment was 32.38% (34/105). Previous studies showed an incidence range of 26–57% during general anesthesia [7, 9–11]. The variance may be possibly attributed to different doses, effect-site concentrations and infusion speeds used in different studies.

The exact mechanisms behind opioid-induced cough remain elusive. One possible mechanism is that opioids cause suppression of central sympathetic outflow and strengthening of the vagus nerve, thus resulting in cough [30]. Pulmonary chemoreflex mediated by irritant receptors or vagal C-fiber receptors close to pulmonary vessels, opioid-induced histamine release from lung mast cells and tracheal smooth muscle constriction may be responsible for occurrence of cough [31]. Control of opioid-induced cough is of paramount clinical significance. Dezocine is a mixed agonist-antagonist opioid and its inhibitory action on opioid-induced cough has been proved in prior studies [17, 32]. In our study, the incidence of remifentanil-induced cough was 32.38% (18 cases of mild cough, 12 cases of moderate cough and 4 cases of severe cough) in the patients pre-administrated with normal saline, and was 6.67% (7 cases of mild cough) in the patients pre-administrated with dezocine. This result revealed that pre-administration of dezocine was effective in suppressing remifentanil-induced cough during general anesthesia induction. In a previous study, no patient pretreated with infusion of dezocine 0.1 mg/kg reports sufentanil-induced cough, in contrast the control patients had a cough incidence of 31.9% [17]. Although dezocine 0.1 mg/kg seems to exert a more potent suppressive effect, higher HR and blood pressure are detected in the patients receiving a pre-emptive infusion of dezocine compared to the control patients in their study. In the current study, 0.03 mg/kg dezocine, a third of an analgesic dose of dezocine was selected. Patients in the dozocine group and the control group were not significantly different in HR and MAP before the anesthesia induction, prior to intubation and 1 min after intubation, and in operating time, 30-min and 60-min postoperative VAS pain scores. These observations indicated that pre-administration of dezocine 0.03 mg/kg does not affect hemodynamics, operating time and postoperative pain of patients. Postoperative analgesia demands a larger dose of dezocine.

Dezocine was thought to be a κ opioid receptor agonist, but recent data suggest it could be a κ opioid receptor antagonist [16]. The cough suppression by dezocine may be due to κ receptors antagonism or decreased norepinephrine and serotonin reuptake [33]. Xu et al. speculated that dezocine may reduce fentanyl-induced cough by antagonizing fentanyl-activated μ receptors via activating κ receptors [34]. Our study did not unveil the exact mechanisms underlying the suppression on remifentanil-induced cough by dezocine. More in-depth studies are necessary to decipher the exact mechanisms. Cough suppressing effect of different doses of dezocine was not explored in the present study. These limitations should be addressed in future studies.

## Conclusion

Our study showed that premedication of a low dose of dezocine was capable of effectively and safely repressing remifentanil-induced cough during general anesthesia in the patients receiving operative hysteroscopy. This study offers more insights concerning alternative regimens to prevent remifentanil-induced cough.

## Data Availability

The datasets used and analyzed in the current study are available from the corresponding author in response to reasonable requests.

## Abbreviations

HR: Heart rate
MAP: Mean arterial pressure
VAS: Visual analog scale
